# Genome-wide survey and expression analysis of *NIN-like Protein* (*NLP*) genes reveals its potential roles in the response to nitrate signaling in tomato

**DOI:** 10.1186/s12870-021-03116-0

**Published:** 2021-07-23

**Authors:** Mengyuan Liu, Xiaona Zhi, Yi Wang, Yang Wang

**Affiliations:** State Key Laboratory of Plant Physiology and Biochemistry, College of Biological Sciences, China Agricultural University, Beijing, 100193 China

**Keywords:** NIN-like Proteins, Tomato, Bioinformatics, Nitrate uptake, Nitrogen deficiency

## Abstract

**Background:**

Tomato (*Solanum lycopersicum*) is one of the most important horticultural crops, with a marked preference for nitrate as an inorganic nitrogen source. The molecular mechanisms of nitrate uptake and assimilation are poorly understood in tomato. NIN-like proteins (NLPs) are conserved, plant-specific transcription factors that play crucial roles in nitrate signaling.

**Results:**

In this study, genome-wide analysis identified six *NLP* members in tomato genome. These members were clustered into three clades in a phylogenetic tree. Comparative genomic analysis showed that *SlNLP* genes exhibited collinear relationships to *NLP*s in Arabidopsis, canola, maize and rice, and that the expansion of the *SlNLP* family mainly resulted from segmental duplications in the tomato genome. Tissue-specific expression analysis showed that one of the close homologs of *AtNLP6/7*, *SlNLP3*, was strongly expressed in roots during both the seedling and flowering stages, that *SlNLP4* and *SlNLP6* exhibited preferential expression in stems and leaves and that *SlNLP6* was expressed at high levels in fruits. Furthermore, the nitrate uptake in tomato roots and the expression patterns of *SlNLP* genes were measured under nitrogen deficiency and nitrate resupply conditions. Four *SlNLPs*, *SlNLP1*, *SlNLP2*, *SlNLP4* and *SlNLP6*, were upregulated after nitrogen starvation. And *SlNLP1* and *SlNLP5* were induced rapidly and temporally by nitrate.

**Conclusions:**

These results provide significant insights into the potential diverse functions of *SlNLPs* to regulate nitrate uptake.

**Supplementary Information:**

The online version contains supplementary material available at 10.1186/s12870-021-03116-0.

## Background

Nitrogen (N), an essential macronutrient for plants, serves as a component of amino acids, nucleotides, chlorophyll, hormones and coenzymes. The growth and development of plants depends on proper nitrogen supply. The availability of N in agricultural fields significantly affects crop yields [26]. Plants absorb inorganic N from the soils mainly in two forms, nitrate (NO_3_^−^) and ammonium (NH_4_^+^). Under mild climatic conditions, nitrate is the main nitrogen source in dry land [10]. The concentration of nitrate in the soils fluctuates between 10 μM and 100 mM [7]. To sustain vigorous growth, high-affinity and low-affinity (*K*_*M*_ > 1 mM) transport systems have been evolved in plants to absorb nitrate efficiently from the environment. Nitrate is also an important signaling molecule for lateral root development, flowering and synergistic absorption of the other nutrients [31].

For nitrate signaling, NIN-like proteins (NLPs) are essential transcription factors [20]. It has been reported that the nutrient-Ca^2+^-NLP regulatory pathway plays a central role in nitrate signaling and integrates transcription, transport, metabolism and systemic growth programs in plants [4, 23, 25]. In *Arabidopsis*, nitrate transporter 1.1 (NPF6.3/NRT1.1) has been identified as a nitrate sensor at the plasma membrane [16]. In the presence of nitrate, calcium-dependent protein kinases 10/30/32 (CPK10/30/32) mediate Ca^2+^ signals by nitrate and phosphorylate NLP6/7 to ensure their location in the nucleus for transcriptional activation of the primary nitrate response genes [23].

NIN protein was first identified in the legume *Lotus japonicus*, with a regulatory function on symbiotic root nodule formation [29]. More NIN proteins and NLPs were found to widely exist among other nonleguminous plants including *Arabidopsis*, rice, wheat, and maize, but not in animals [21, 27, 30, 33]. Both NIN proteins and NLPs have a RWP-RK domain for DNA binding; NLPs carry an additional PB1 domain for protein–protein interactions [5]. Interactions between NLPs and other transcription factors such as nitrate regulatory gene 2 (NRG2) [34], PCF (TCP)-domain family protein 20 (TCP20) [14], and nitrate-inducible GARP-type transcriptional repressor 1 (NIGT1) [24] have been reported. Beyond nitrate signaling, extra functions of NLPs in the N starvation response [14], N and phosphate (P) interactions [24], nitrate-promoted seed germination [35], nitrate-dependent nodule symbiosis [28] and root cap cell release [19] have been clarified.

As one of the most important crops, tomato (*Solanum lycopersicum*) shows a marked preference for nitrate as an inorganic nitrogen source [8].

In the present study, comparative bioinformatics analysis of the tomato *NLP* genes was performed. Furthermore, the rate of root nitrate uptake and the expression of *SlNLP* genes under nitrogen deficiency and nitrate resupply conditions were detected to evaluate their potential roles in nitrate uptake regulation in roots.

## Results

### Identification of *NLP* Genes in tomato

A total of six *NLP* genes were identified from the tomato genome based on the presence of conserved RWP-RK (hmm, PF02042) and PB1 domains (hmm, PF00564). The nomenclature used for *SlNLP* genes was based on their distribution on the chromosomes (Table 1). The numbers of amino acids coded by *SlNLP* genes ranged from 841 (*SlNLP1*) to 1611 (*SlNLP5*). The relative molecular weights (Mw) were between 93.30 kDa (SlNLP1) and 180.95 kDa (SlNLP5). All SlNLP proteins had an isoelectric point near neutral (5.30–7.35), and low hydrophilicity indicated by GRAVY values (− 0.524 to − 0.327). The subcellular localizations were predicted to be in the nucleus/cytosol for all six SlNLPs.

**Table 1:** Identification of NLP Genes in tomato

Gene Name	Gene ID	Protein characteristics				Subcellular localization
		Length (aa)	Mw (Da)	pI	GRAVY	
*SlNLP1*	Solyc01g112190.3	841	93,298.51	7.35	-0.524	Nucleus/cytosol
*SlNLP2*	Solyc04g082480.3	912	102,467.99	5.58	-0.520	Nucleus/cytosol
*SlNLP3*	Solyc08g008410.3	1008	109,783.94	5.70	-0.327	Nucleus/cytosol
*SlNLP4*	Solyc08g013900.3	961	106,149.69	5.41	-0.347	Nucleus/cytosol
*SlNLP5*	Solyc08g082750.3	1611	180,948.88	6.16	-0.473	Nucleus/cytosol
*SlNLP6*	Solyc11g045350.2	986	108,349.29	5.30	-0.416	Nucleus/cytosol

*Mw* molecular weight, *pI* isoelectric point, *GRAVY* grand average of hydropathicity

### Conserved motifs and phylogenetic analysis of *SlNLP* proteins

**Fig. 1:**
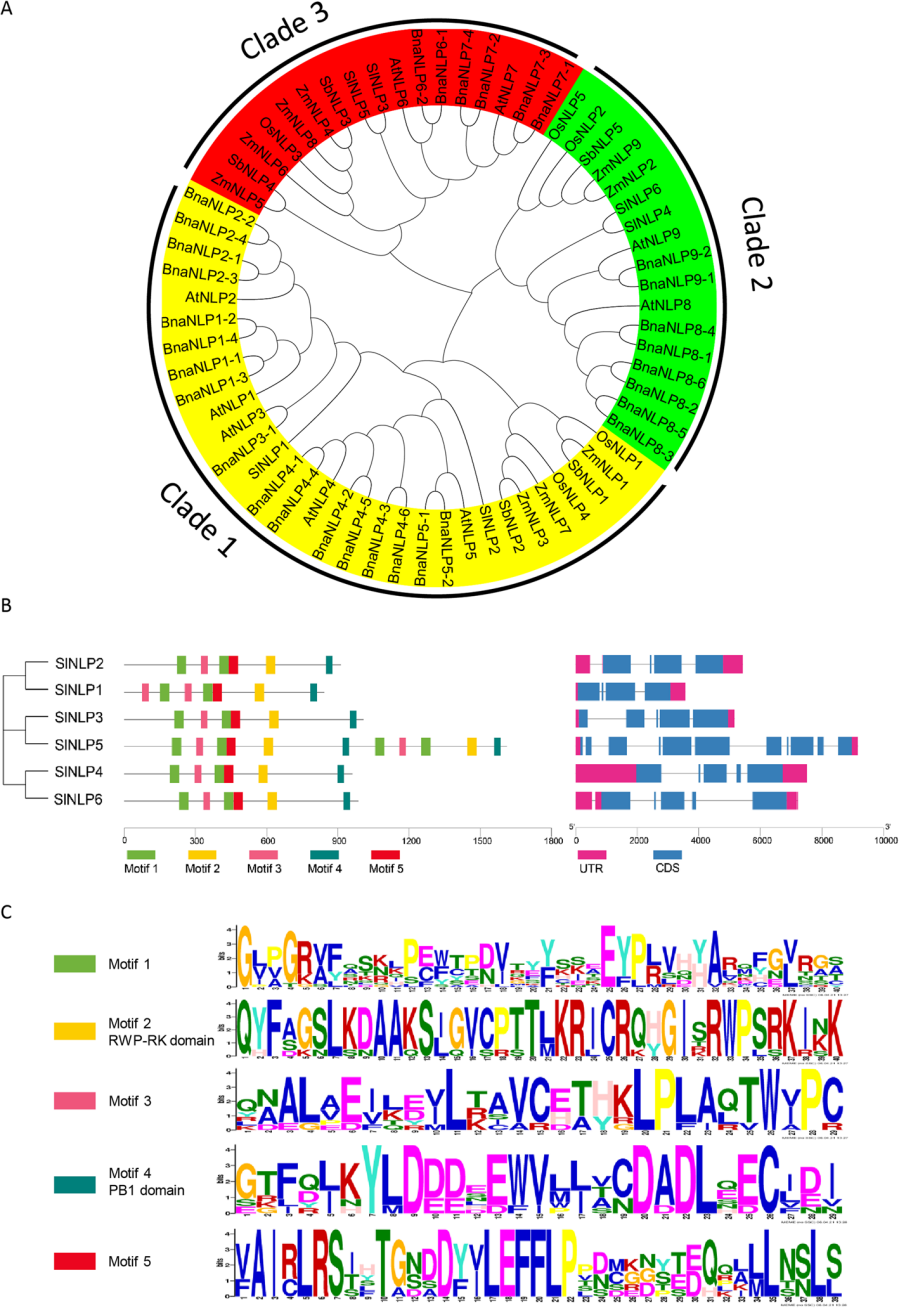
Phylogenetic tree and conserved motifs of the *NLP* gene family. **A** A neighbor-joining phylogenetic tree of NLPs from tomato (*Solanum lycopersicum*), Arabidopsis (*Arabidopsis thaliana*), canola (*Brassica napus*), rice (*Oryza sativa*) and maize (*Zea mays*). All NLP proteins were assigned into three clades. **B** Motifs were identified by MEME. The motifs are displayed in different colors. The scale bar represents 300 amino acids. The exon-intron structures were identified by GSDS. The untranslated regions (UTR) and coding sequences (CDS) are displayed in different colors. The scale bar represents 2000 nucleotides. **C** Sequences of identified motifs including three unknown domains (yellow, pink and red), the RWP-RK domain (yellow) and the PB1 domain (dark green)

Based on a previous study, *Arabidopsis* NLP proteins were divided into three clades [30]. To analyze the evolutionary relationship of tomato NLP proteins, a neighbor-joining phylogenetic tree was constructed by comparing tomato NLP amino acid sequences with NLPs from four other plant species, including two dicotyledonous plants (*Arabidopsis* and canola) and two monocotyledonous plants (rice and maize) (Supplementary Table 1). The results (Fig. 1A) showed that Clade I contained 17 NLP members, including AtNLP1/2/3/4/5 and SlNLP1/2. Clade II contained 17 NLP members, including AtNLP6/7 and SlNLP3/5. Clade III contained 31 NLP members, including AtNLP8/9 and SlNLP4/6. Both dicotyledonous and monocotyledonous members existing in every clade indicated that gene expansion of the *NLP* gene family occurred before the ancestral divergence of monocotyledons and dicotyledons. Multiple sequence alignment (Fig. 1B and C) revealed that all the SlNLP proteins share similar motif patterns, including the conserved RWP-RK domain and PB1 domain. Interestingly, the SlNLP5 protein appeared to carry double RWP-RK domains and PB1 domains.

### Chromosomal distribution and syntenic analysis of *SlNLP* genes

**Fig. 2:**
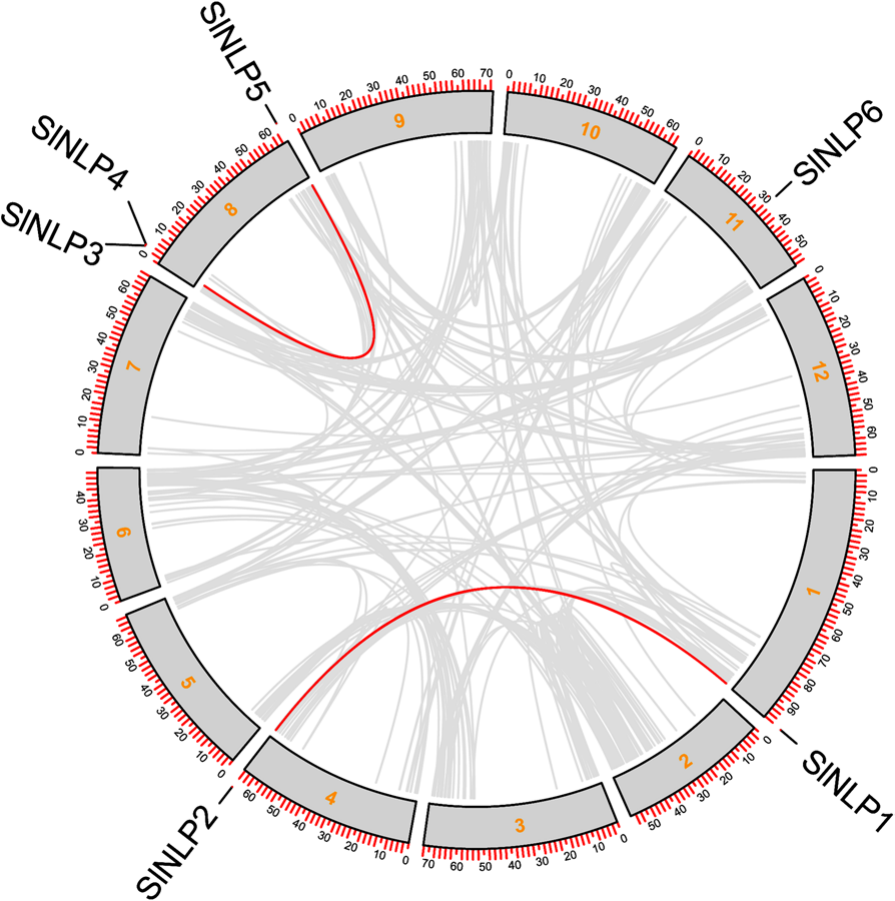
Chromosomal distribution and interchromosomal relationship of tomato *NLP* genes. The inner-species collinearity of *SlNLPs*. Gray lines indicate all syntenic blocks in the tomato genome, and the red lines indicate the duplicated *SlNLP* gene pairs. The number in the gray box area is the chromosome number

Six *SlNLP* genes were distributed unevenly in the tomato genome (Fig. 2). *SlNLP3*, *SlNLP4* and *SlNLP5* were identified on chromosome 8. The other three *SlNLP* genes, *SlNLP1*, *SlNLP2* and *SlNLP6* genes were identified on chromosomes 1, 4 and 11, respectively. The interchromosomal relationship of *SlNLP* genes showed two pairs of segmental duplications (*SlNLP1* and *SlNLP2*, *SlNLP3* and *SlNLP5*), indicating that tomato *NLP* genes were mainly generated by gene duplication during evolution.

**Fig. 3:**
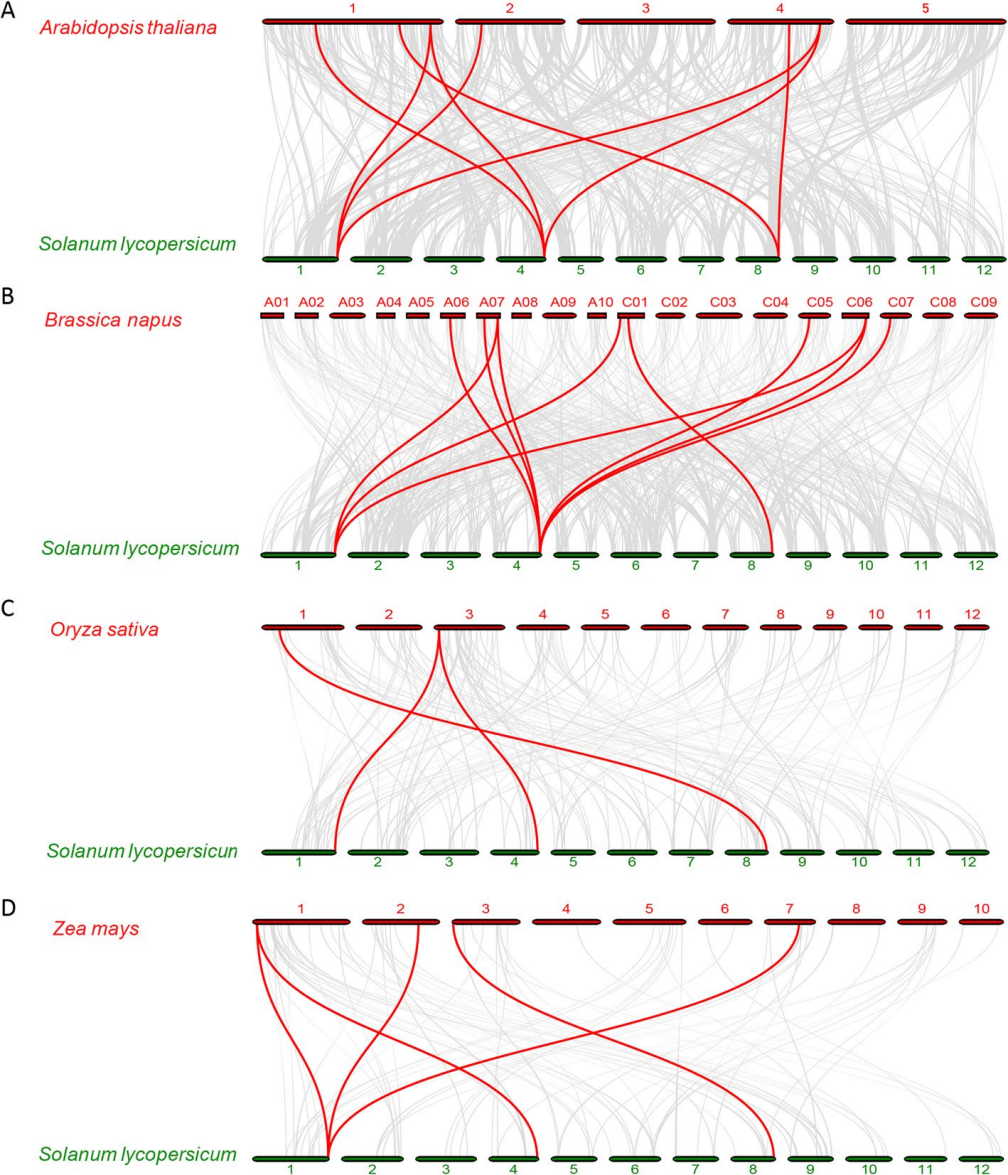
Syntenic *NLP* gene pairs between tomato (*Solanum lycopersicum*) and four other plant species, including **A** *Arabidopsis thaliana*; **B** *Brassica napus*; **C** *Oryza sativa*; **D** *Zea mays*. Gray lines indicate all the collinear blocks in the genome, and the red lines indicate the syntenic *NLP* gene pairs

Furthermore, four comparative syntenic maps between tomato and *Arabidopsis*, canola, rice and maize, separately, were constructed to analyze the phylogenetic mechanisms of *SlNLPs* (Fig. 3). Tomato *SlNLP* genes showed 10 syntenic gene pairs with canola, 8 with *Arabidopsis*, 5 with maize and 3 with rice. Most background collinear blocks associated with *NLP* gene pairs identified between tomato and dicotyledon *Arabidopsis*/canola contained more genes than those between tomato and monocotyledon rice/maize (Supplementary Table 2). *SlNLP1*, *SlNLP2* and *SlNLP5* were found in the four comparative syntenic maps, suggesting that these orthologous pairs might already exist before evolutionary divergence of monocotyledons and dicotyledons. In addition, these three genes might have played fundamental roles in the *NLP* gene family. The ratio of nonsynonymous (Ka) to synonymous substitutions (Ks), presenting the selection type acting on the coding sequences, was also calculated (Supplementary Table 2). Two *SlNLP* gene pairs, *SlNLP1* and *SlNLP2*, as well as *SlNLP3* and *SlNLP5*, had Ka/Ks ratios of 1.01 and 1.46, respectively, indicating positive selection during evolution for functional divergence occurring after duplication. Most of the orthologous *NLP* gene pairs had a Ka/Ks ratio less than 1 (ranging from 0.10 to 0.96), suggesting purifying selective pressure during *NLP* gene family evolution and conserved functions of these genes. Three orthologous gene pairs, *SlNLP1* and *AtNLP5*, *SlNLP2* and *BnaNLP4-4*, *SlNLP1* and *ZmNLP1*, had a Ka/Ks ratio greater than 1, indicating that these genes have undergone positive selection pressure and might have evolved new functions to help plants cope with their living environments.

### Organ-dependent expression of *SlNLPs*

**Fig. 4:**
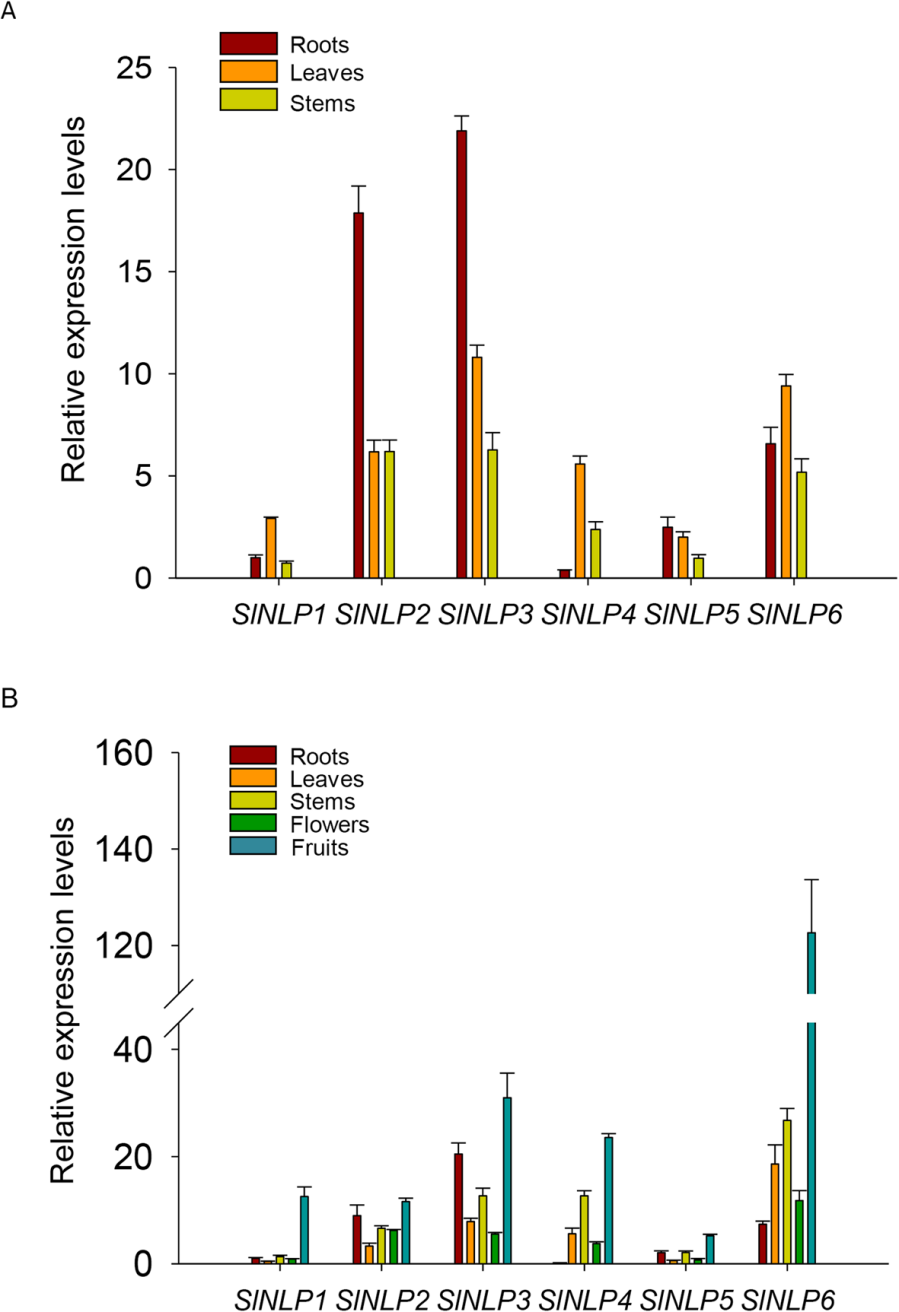
Tissue-specific expression of *SlNLPs*. **A** Relative expression levels of *SlNLPs* in roots, leaves and stems at the seedling stage; **B** Relative expression levels of *SlNLPs* in roots, leaves, stems and flowers at the flowering stage and in red fruits. Gene expression levels were normalized to the *SlEF1a* gene. And *SlNLP1* expression levels in roots were set to 1. Data shown as mean ± s.d. of four independent biological replicates

To obtain evidence of physiological function, the tissue-specific transcript abundance of six *SlNLP* genes was analyzed by qRT-PCR at different developmental stages (Fig. 4). *SlNLP1* expression levels in roots were set to 1 for comparison of expression levels of *SlNLPs*. At both the seedling and flowering stages, *SlNLP2* and *SlNLP3* were preferentially expressed in roots (Fig. 4A and B). *SlNLP2* and *SlNLP3* showed the highest transcript abundance in roots at the seedling stage (Fig. 4A). When flowering, *SlNLP3* still showed the highest abundance in roots, followed by *SlNLP2* and *SlNLP6* (Fig. 4B). In the red fruits, the transcript abundance of all the *SlNLP* genes was in the relatively high level. Interestingly, *SlNLP6* exhibited increasing transcript accumulation in all the test tissues after flowering. And in particular, significantly higher *SlNLP6* expression was observed in fruits (Fig. 4B).

### Expression of* SlNLPs* in response to nitrogen deficiency

**Fig. 5:**
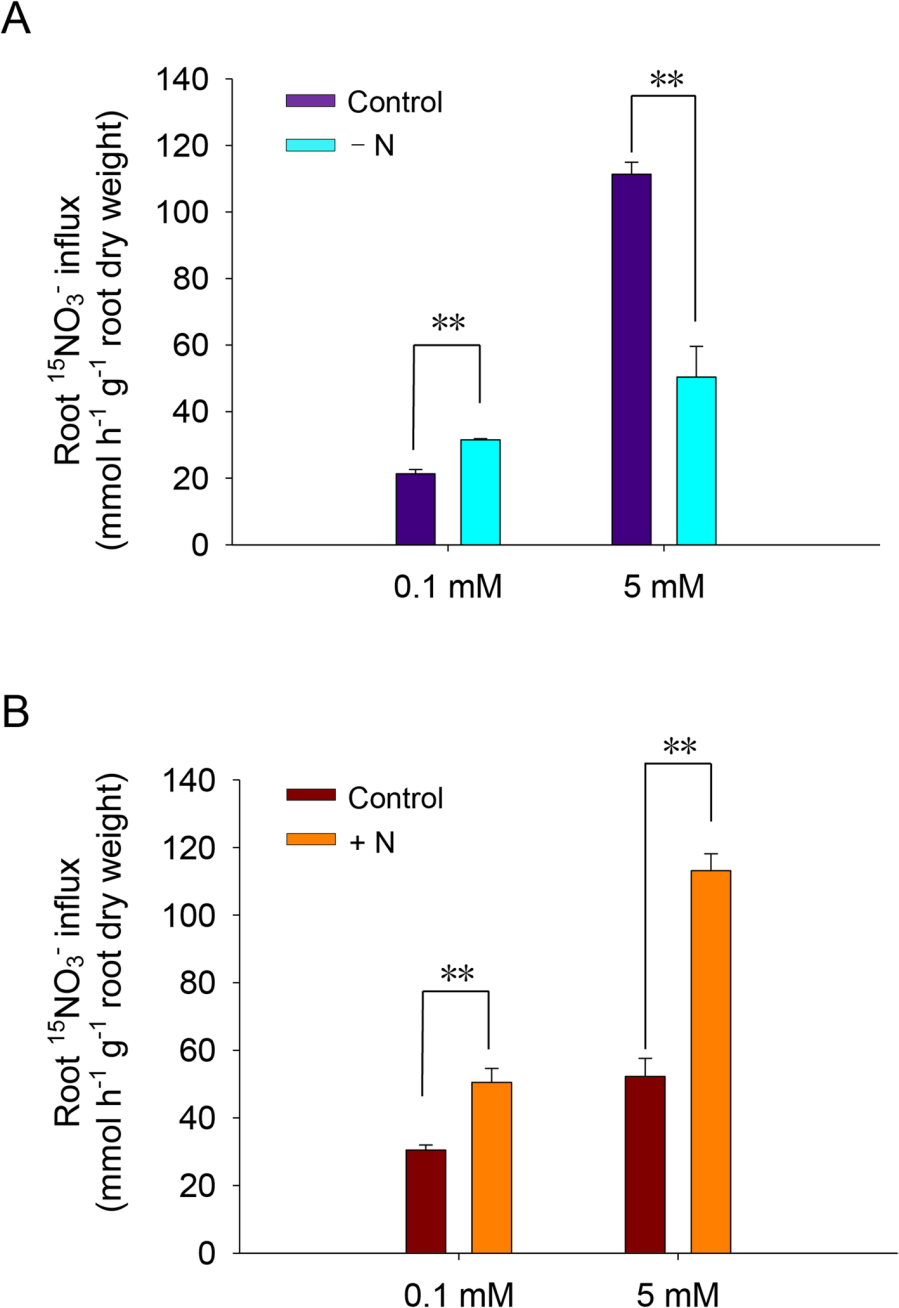
Root high-affinity and low-affinity ^15^NO_3_^–^ uptake assay under nitrogen starvation and nitrate induction. **A** Seedlings were treated with nitrogen starvation (-N) for 2 days. Seedlings grown in normal hydroponic medium were used as the control; **B** Nitrogen-starved seedlings were then resupplied with 5 mM KNO_3_ for 2 h. Nitrogen-starved seedlings resupplied with 5 mM KCl were used as the control. The root high-affinity and low-affinity ^15^NO_3_^–^ uptake abilities were detected in 0.1 mM or 5 mM K^15^NO_3_ solution, respectively, for 5 min. Data shown as mean ± s.d. of three independent biological replicates, ** *p* < 0.01

**Fig. 6:**
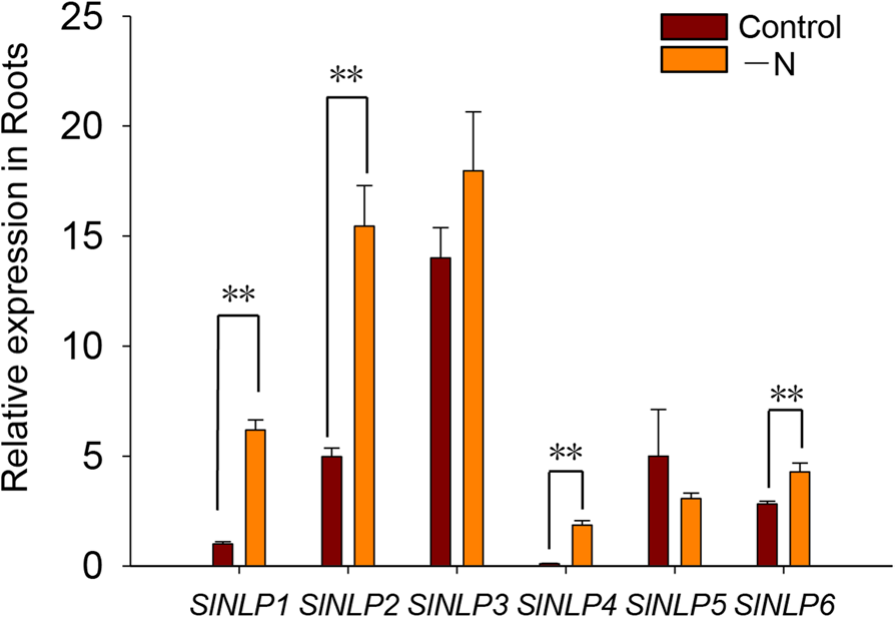
Expression of *SlNLPs* in response to nitrogen deficiency. Seedlings were treated with nitrogen starvation (-N) for 2 days. Total RNA was extracted from roots after treatment and subjected to qRT-PCR analysis. Gene expression levels were normalized to the *SlEF1a* gene. And *SlNLP1* expression level in normal hydroponic medium was set to 1. Data shown as mean ± s.d. of four independent biological replicates, ** *p* < 0.01

Nitrate absorption in tomato roots was found to be influenced by two-days’ nitrogen starvation treatment, as indicated by the ^15^NO_3_^−^ influx assay after different treatments (Fig. 5A). The results showed that the root high-affinity nitrate uptake ability was enhanced under nitrogen starvation, but root low-affinity nitrate uptake ability was repressed. To obtain evidence of possible roles of SlNLPs in root nitrate absorption regulation during nitrogen deficiency, the transcript abundance of *SlNLP* genes in roots was examined by qRT-PCR after starvation treatments (Fig. 6). The expression of *SlNLP1, SlNLP2, SlNLP4* and *SlNLP6* was upregulated 6.2-, 3.1-, 17- and 1.5-fold, respectively, after nitrogen starvation.

### Nitrate-dependent expression of* SlNLPs* and nitrogen metabolism genes

**Fig. 7:**
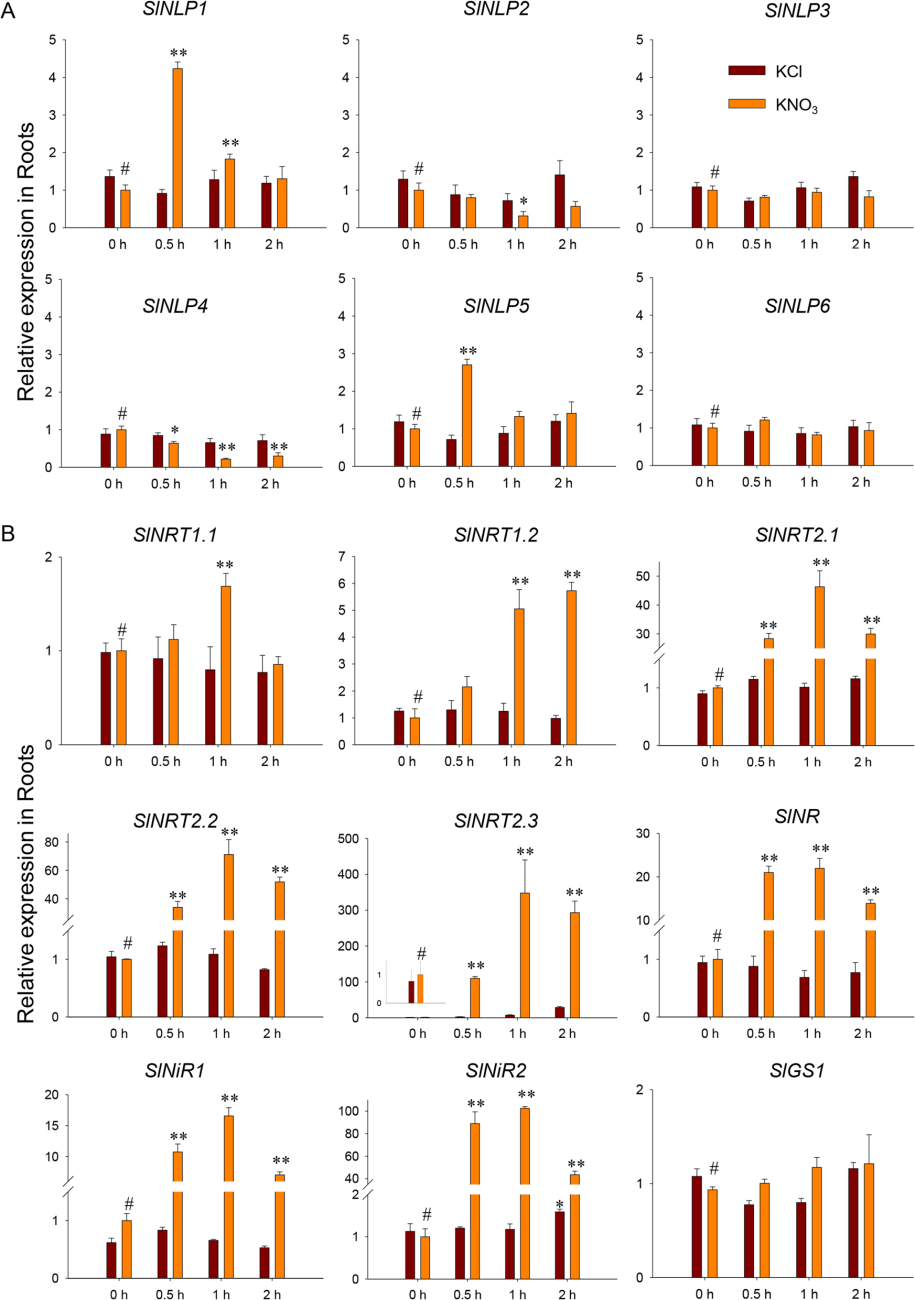
Nitrate-dependent gene expression. Seedlings were nitrogen-starved for 2 days and resupplied with 5 mM nitrate or 5 mM KCl as a control for 2 h. Total RNA was extracted from roots at 0, 0.5, 1 and 2 h after treatment and subjected to qRT-PCR analysis. **A** *SlNLP* genes; **B** nitrate transport- and metabolism-associated genes. Gene expression levels were normalized to the *SlEF1a* gene, and the expression level in samples at 0 h in 5 mM KCl medium was set to 1. Data shown as mean ± s.d. of four independent biological replicates. “#” represents the control, ** *p* < 0.01 and * *p* < 0.05

When nitrate was resupplied to the nitrogen-starved seedlings, both the root high-affinity and low-affinity nitrate uptake rates were enhanced, as shown by the results of the ^15^NO_3_^−^ influx assay (Fig. 5B). The expression levels of *SlNLPs* and nitrogen metabolism genes in roots were examined at 0.5, 1 and 2 h during the nitrate induction process. The results (Fig. 7A) showed that the transcript abundance of *SlNLP1* and *SlNLP5* increased rapidly and temporally in response to nitrate. *SlNLP1* and *SlNLP5* expression reached the maximum levels (4.1- and 2.8-fold increases, respectively) 0.5 h after nitrate was supplied. The expression of *SlNLP2* and *SlNLP4* was repressed significantly after nitrate resupply for 1 h. In contrast, *SlNLP3* and *SlNLP6* did not show any response to nitrate at the transcription level. The transcript abundance of the nitrate transporters and nitrate assimilation genes is presented in Fig. 7B. The expression of the high-affinity nitrate transporter genes *SlNRT2.1*, *SlNRT2.2* and *SlNRT2.3*, and the nitrate reductase gene *SlNR* and nitrite reductase genes *SlNiR1* and *SlNiR2* increased rapidly and violently within the first 30 min of exposure to nitrate, and remained at very high levels. The expression level of the low-affinity nitrate transporter gene *SlNRT1.2* increased to twofold at 0.5 h after nitrate resupply and further increased by greater than fourfold at 1 and 2 h. Minimal stimulation of transcription of another low-affinity nitrate transporter gene, *SlNRT1.1*, was demonstrated by an temporary 1.6-fold increased at 1 h after nitrate resupply. No mRNA expression change in the glutamine synthetase gene *SlGS* was detected during the 2-h nitrate induction period. Protein interaction networks of SlNLP proteins were predicted (Supplementary Figure 1). SlNLP3, SlNLP4, SlNLP5 and SlNLP6 showed potential interactions with nitrate reductase SlNR. SlNLP3 and SlNLP5 showed additional potential interactions with nitrite reductases SlNiR1 and SlNiR2 implying their central role in nitrate responses. SlNLP1 and SlNLP2 displayed major interactions with the transcription factor GRAS16, indicating they might act as regulators associated with plant development.

## Discussion

In the present study, genome-wide analysis revealed six tomato *NLPs* (Table 1). The *NLP* family size of *Solanum lycopersicum* is similar to that of *Arabidopsis thaliana* (9), *Oryza sativa* (5) and *Zea mays* (9), and much smaller than that of *Brassica napus* (31). Phylogenetic analysis showed that every *NLP* family has members belonging to three groups (Fig. 1A). All of SlNLPs have conserved RWP-RK and PB1 domains. SlNLP5 is special for double RWP-RK and PB1 domains (Fig. 1B). The expansion of the tomato *NLP* gene family was mainly generated by gene duplication in the genome (Fig. 2). Orthologous gene pairs associated with *SlNLP1, SlNLP2* or *SlNLP5* were indicated to have existed before the ancestral divergence of dicotyledonous and monocotyledonous plants (Fig. 3). It is worth noting that the Ka/Ks ratios of two paralogous *SlNLP* gene pairs (*SlNLP1* and *SlNLP2*, *SlNLP3* and *SlNLP5*) and three orthologous *NLP* gene pairs (*SlNLP1* and *AtNLP5*, *SlNLP2* and *BnaNLP4-4*, *SlNLP1* and *ZmNLP1*) were greater than 1 (Supplementary Table 2), representing positive selection and fast evolutionary rates in these SlNLPs at the protein level. Therefore, it is implied that *NLPs* in tomato might have evolved some new functions to meet their growth and development demands of the plant.

Tissue-dependent expression patterns showed that all 6 *SlNLP* genes were expressed in all tested tissues including roots, stems, leaves, flowers and fruits (Fig. 4), which is similar to *NLPs* in Arabidopsis [5], maize (Ge et al. [13]) and *Brassica napus* [5]. *SlNLP3*, one of the close homologs of *AtNLP6/7* (Fig. 1A), the key component of nitrate signaling [23], has the highest expression level in roots at both the seedling and flowering stages. In addition to *SlNLP3*, *SlNLP2* and *SlNLP6* were also expressed at high levels in roots, at different stages of development, implying their different functions in nitrate uptake regulation, rather than simple functional redundancy. Two *SlNLPs* from Clade III, *SlNLP4* and *SlNLP6*, were preferentially expressed in aboveground tissues and their transcriptional abundance was strongly upregulated during flowering, suggesting that they might probably regulate nitrogen translocation and assimilation to support flower and fruit development. In contrast to *SlNLP4*, *SlNLP6* had higher transcript abundance in both roots and aboveground tissues. Moreover, *SlNLP6* showed extremely higher expression levels than all the other five *SlNLPs* in fruits (Fig. 4B). Interestingly, *SINLP6* also showed its uniqueness in syntenic analysis between *NLP* genes (Fig. 2). The close homolog of *SlNLP6* is *AtNLP8* (Fig. 1A). AtNLP8 has been reported as a master regulator of nitrate-promoted seed germination [35], which might provide some hints for functional roles of *SlNLP6* in fruits*.*

As one of the fundamental regulatory elements at the transcriptional level, NLPs play important roles in nitrate uptake and assimilation regulation [11, 15]. In *Arabidopsis*, *nlp7* mutants show features of a nitrogen-starved plant [4],*AtNLP7* overexpression increases plant biomass under both nitrogen-poor and nitrogen-rich conditions (Yu et al*.* [36]). Expression of rice *NLPs* (*OsNLP1*, *OsNLP4* and *OsNLP5*) was promoted by nitrogen deficiency as well as nitrate supply [18]. Overexpression of *OsNLP1* could enhance rice nitrogen use efficiency [2]. For tomato, nitrate is a more favorable inorganic nitrogen source form. Nitrate uptake in tomato roots is under precise regulation with complex interactions between nitrogen and the other essential macronutrients phosphate and/or potassium availability [31]. When the environmental nitrogen source was depleted, the root low-affinity nitrate influx rate decreased, but the high-affinity nitrate influx rate increased (Fig. 5A). Similar results have been reported: higher nitrate influx was detected in tomatoes growing in nutrient solutions containing 5 mM nitrate than in tomatoes growing in nutrient solutions containing 0.1 mM nitrate [1]. Both low-affinity and high-affinity nitrate uptake in roots increased after nitrate was resupplied to the nitrogen-starved tomato seedlings (Fig. 5B). The question is whether some SlNLPs play the important roles in nitrogen absorption regulation during nitrogen starvation and/or nitrate induction.

To answer this question, the transcript abundance of *SlNLPs* in roots was detected under nitrogen deficiency (Fig. 6) and nitrate resupply (Fig. 7A). Most of *SlNLPs* (*SlNLP1, SlNLP2, SlNLP4* and *SlNLP6*) showed upregulated expression after nitrogen starvation for 2 days. When nitrate was resupplied, the temporal expression of *SlNLP2* and *SlNLP4* was repressed, but *SlNLP1* was still showed rapidly upregulated. SlNLP3, which showed the highest expression level in roots during both seedling and flowering stages (Fig. 4), had potential interactions with nitrate reductase and nitrite reductase (Supplementary Figure 1). These results imply the central role of SlNLP3 in nitrate responses. However, *SlNLP3* did not show any response to nitrate in the transcriptional level (Fig. 7A). The other close homolog of *AtNLP6/7*, *SlNLP5*, showed little transcriptional response to nitrogen starvation but was induced rapidly and temporally by nitrate. It is noteworthy that AtNLP6/7 responds to nitrate signaling not at the transcriptional level [23]. It is possible that a similar situation also exists in tomato. Therefore, the protein levels and protein modifications (including phosphorylation) of SlNLPs should be examined. It is interesting to determine how SlNLP3 participates in nitrogen deficiency response and/or nitrate signaling pathway in the future.

## Conclusions

In summary, this study provided a genome-wide analysis of *NLP* genes in tomato. *NLP* genes are highly conserved among tomato, *Arabidopsis*, canola, maize and rice. Segmental duplication was the major driving force of *SlNLP* gene evolution. Some *SlNLP* genes have undergone positive selection during evolution, probably leading to functional divergence in gene families. The expression patterns of *SlNLP* genes provide hints for their diverse physiological roles in tomato growth and development, especially in nitrate uptake regulation. Further functional analysis for each *SlNLP*, especially *SlNLP3* and *SlNLP6*, will be necessary to explore their regulatory functions. It is believed that a comprehensive understanding of the roles of *SlNLP* under fluctuating nutrition conditions is an essential step towards deciphering the molecular mechanism of nitrogen utilization and promoting nitrogen use efficiency in tomato.

## Methods

### Database search for NLP proteins

Raw hidden Markov model (HMM) data of the conserved RWP-RK (PF02042) and PB1 (PF00564) domains downloaded from Pfam (http://pfam.xfam.org) [9] were used to search for their orthologs in the tomato genome (Solanum_lycopersicum.SL3.0), with an e-value of less than 1e − 10 in Phytozome (https://phytozome-next.jgi.doe.gov/info/Slycopersicum_ITAG2_4). Then, the results were confirmed by based on the SMART (http://smart.embl.de/), NCBI Conserved Domains Database (CDD) (http://www.ncbi.nlm.nih.gov/cdd), and Plant Transcription Factor Database (TFDB) (http://planttfdb.cbi.pku.edu.cn/) databases. The physicochemical properties of SlNLP proteins, including peptide length (aa), molecular weight (Mw), isoelectric point (pI) and grand average of hydrophilicity (GRAVY) were predicted using ExPASy ProtParam (http://web.expasy.org/protparam/) [12]. Subcellular localizations of SlNLP proteins were predicted using CropPAL2020 (https://www.crop-pal.org) [17].

### Multiple sequences alignment and phylogenetic analysis

Clustal W (version 2.1) was employed for the multiple sequence alignment and sequence identity matrix of the proteins [22]. Then, the deduced amino acid sequences in the RWP-RK and PB1 domains were adjusted manually using GeneDoc software. A phylogenetic tree was constructed with the MEGAX program (http://www.megasoftware.net/) using the neighbor-joining method. Proportions of amino acid differences were computed using Poisson correction distances to estimate evolutionary distances. The pairwise deletion option was used to circumvent the gaps and missing data. The conserved protein motifs of SlNLP proteins were analyzed using MEME server v5.3.0 (http://meme-suite.org/tools/meme) [3]. The parameters for the search were as follows: the max motif number to find is 5, and min–max motif width is 2–40. The matched motifs with low quality were manually removed based on an e-value of less than 1e − 15. The exon-intron structures of the *SlNLP* genes were identified on the Gene Structure Display Server (GSDS 2.0, http://gsds.gao-lab.org/). Sequences of NLP proteins of tomato (*Solanum lycopersicum*), *Arabidopsis* (*Arabidopsis thaliana*), canola (*Brassica napus*), rice (*Oryza sativa*) and maize (*Zea mays*) were downloaded from Phytozome (https://phytozome.jgi.doe.gov/).

### Chromosomal distribution and gene duplication

Chromosome distribution and gene duplication events were analyzed using the Multiple Collinearity Scan toolkit MCScanX. The syntenic analysis maps of orthologous *NLP* genes were constructed using the Dual Systeny Plotter software (https://github.com/CJ-Chen/TBtools) [6]. Nonsynonymous (Ka) and synonymous (Ks) substitutions of each duplicated *NLP* gene were calculated using KaKs_Calculator 2.0 [32].

### Analysis of protein–protein interaction networks

To study the protein–protein interaction network, SlNLP protein sequences were analyzed in Ensembl Database SL3.0 (http://plants.ensembl.org/index.html) followed by prediction of interaction partners and networks using the STRING tool (http://string-db.org/).

### Plant materials and treatments

Tomato ecotype Micro-Tom was used in this study. The seeds were germinated and grown on vermiculite for 7 d before transfer to hydroponics. The hydroponic minimal medium comprised 2 mM KH_2_PO_4_, 2 mM MgSO_4_, 25 μM H_3_BO_3_, 2 μM ZnSO_4_, 2 μM MnCl_2_, 0.5 μM CuSO_4_, 0.5 μM Na_2_MoO_4_, and 20 μM Fe-EDTA. This medium was supplemented with 1.3 mM Ca(NO_3_)_2_, 1.5 mM KNO_3_, 0.14 mM KH_2_PO_4_, and 1 mM MgSO_4_ under normal conditions. The pH of the solutions was maintained at approximately 5.8. Nutrient solutions were completely replaced weekly. Plants were grown at 28/22 °C with a 16/8 h light/dark photoperiod. Plants grown in hydroponics for 4 weeks were used for nitrogen starvation treatment and nitrate induction treatment. For nitrogen starvation treatment (-N), hydroponic minimal medium with 1 mM CaCl_2_, 0.6 mM K_2_SO_4_, 0.25 mM KH_2_PO_4_, and 0.5 mM MgSO_4_ was used for 2 days. For nitrate induction treatment, N-starved plants were resupplied with 5 mM nitrate medium (hydroponic minimal medium with KNO_3_) for the indicated time.

### RNA extraction, cDNA synthesis, and qRT-PCR

Total RNA of different tissues was extracted using M5 SuperPure Total RNA Extraction Reagent (Mei5 Biotechnology Co. Ltd). Then, the DNA-free RNA was used to synthesize cDNA by using a RevertAid First Strand cDNA Synthesis Kit (Cat. No. K1622, Thermo). Quantitative RT-PCR (qRT-PCR) was performed using a SYBR Green PCR Master Mix (Life Technologies) in 7500 Real-Time PCR System (Applied Biosystems). Relative expression levels of *SlNLPs* were examined at the seedling stage, at the flowering stage and in red fruits. The housekeeping tomato *EF1*a gene (*Solyc06g009970.3*) was used as an internal control. Primer sequences used for qRT-PCR are listed in Supplementary Table 3.

### ^15^NO_3_^−^ uptake assay

^15^NO_3_^−^ influx in roots was determined as previously described [37]. Tomato roots were washed in 0.1 mM CaSO_4_ for 1 min and then submerged in medium containing 1 mM or 5 mM K^15^NO_3_ (atom% ^15^ N: 99%) for 5 min and finally in 0.1 mM CaSO4 for 1 min. Roots were separated from the shoots immediately after the final transfer to CaSO_4_, and frozen in liquid nitrogen. After grinding, an aliquot of the frozen powder was dried overnight at 80 °C. The ^15^N concentration was measured using an isotope ratio mass spectrometer (IRMS; DELTA^plus^ XP). The influx of ^15^NO_3_^−^ was calculated from the ^15^N content of the roots (1 mg DW).

### Statistical analysis

Data were processed using the statistics program SPSS version 21. The statistical significance of differences in ^15^N influx and gene expression was examined by Student’s t-test (* *p* < 0.05, ** *p* < 0.01).

## Supplementary Information

**Additional file 1: Supplementary Table 1.** *NLP* genes from tomato, Arabidopsis, canola, rice and maize.

**Additional file 2: Supplementary Table 2.** One-to-one orthologous relationships between tomato and the other four plant species.

**Additional file 3: Supplementary Table 3.** Primers used in qRT-PCR.

**Additional file 4: Supplementary Figure 1.** Protein-protein interaction network of SlNLP proteins.

## Data Availability

The databases used in the study includes Pfam (http://pfam.xfam.org), Phytozome (https://phytozome.jgi.doe.gov/), SMART (http://smart.embl.de/), NCBI Conserved Domains Database (CDD) (http://www.ncbi.nlm.nih.gov/cdd), Plant Transcription Factor Database (TFDB) (http://planttfdb.cbi.pku.edu.cn/), ExPASy ProtParam (http://web.expasy.org/protparam/), CropPAL2020 (https://www.crop-pal.org), MEGAX (http://www.megasoftware.net/), MEME server v5.3.0 (http://meme-suite.org/tools/meme), Dual Systeny Plotter software (https://github.com/CJ-Chen/TBtools), Ensembl Database SL3.0 (http://plants.ensembl.org/index.html), STRING (http://string-db.org/). The public access to all these databases is open. The datasets used and/or analyzed during the current study available from the corresponding author on reasonable request.

## Abbreviations

NLPs: NIN-like proteins
NRTs: Nitrate transporters
