# Supplementary material for: Genome-wide survey and expression analysis of NIN-like Protein (NLP) genes reveals its potential roles in the response to nitrate signaling in tomato
Source: BMC Plant Biol. 2021 Jul 23;21:347. doi: 10.1186/s12870-021-03116-0 (PMC8299697; doi:10.1186/s12870-021-03116-0)
Supplement: Supplementary file 4 — Additional file 4: Supplementary Figure 1. Protein-protein interaction network of SlNLP proteins. [file 12870_2021_3116_MOESM4_ESM.pdf]

Supplementary Fig. 1
Protein-protein interaction network of SlNLP proteins.

SlNLP1

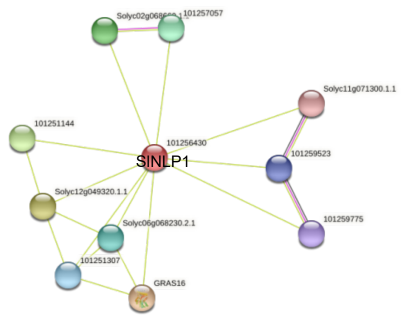

node	annotation
GRAS16	Belongs to the GRAS family
Solyc12g049320.1.1	No descriptions
101251144	Belongs to the GRAS family
Solyc02g068660.1.1	No descriptions
101257057	No descriptions
101251307	No descriptions
101259523	DP-E2F-like 1
101259775	No descriptions
Solyc11g071300.1.1	No descriptions
Solyc06g068230.2.1	Tetratricopeptide repeat (TPR)-like superfamily protein

SlNLP2

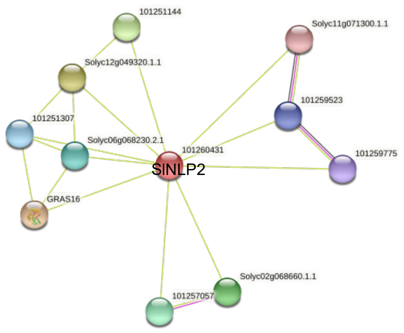

node	annotation
GRAS16	Belongs to the GRAS family
Solyc12g049320.1.1	No descriptions
101251144	Belongs to the GRAS family
Solyc02g068660.1.1	No descriptions
101257057	No descriptions
Solyc06g068230.2.1	Tetratricopeptide repeat (TPR)-like superfamily protein
101251307	No descriptions
101259523	DP-E2F-like 1
101259775	No descriptions
Solyc11g071300.1.1	No descriptions

SlNLP3

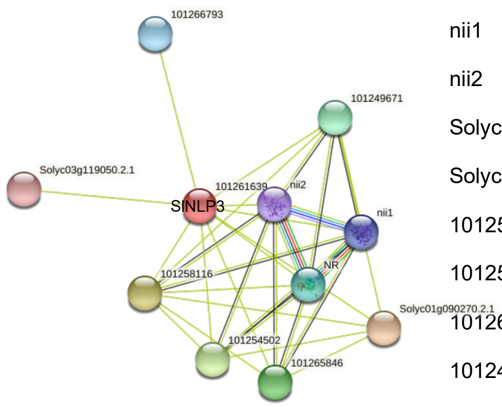

node	annotation
NR	Nitrate reductase
nii1	Nitrite reductase
nii2	Nitrite reductase
Solyc03g119050.2.1	Myb domain protein 4r1
Solyc01g090270.2.1	Protein of unknown function
101258116	No descriptions
101254502	No descriptions
101265846	No descriptions
101249671	No descriptions
101266793	No descriptions

Supplementary Fig. 1 continued
Protein-protein interaction network of SINLP proteins.

SINLP4

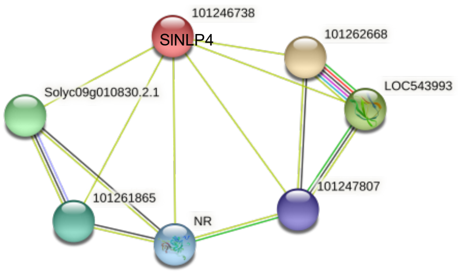

node	annotation
NR	Nitrate reductase
101262668	3-methylcrotonyl-CoA carboxylase
LOC543993	Biotin carboxyl carrier protein of acetyl-CoA carboxylase
Solyc09g010830.2.1	No descriptions
101261865	No descriptions
101247807	No descriptions

SINLP5

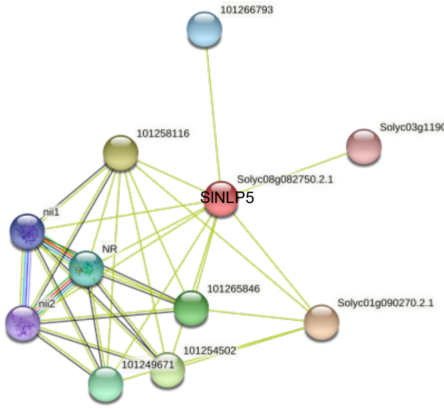

node	annotation
NR	Nitrate reductase
nii1	Nitrite reductase
nii1	Nitrite reductase
Solyc01g090270.2.1	No descriptions
Solyc03g119050.2.1	Myb domain protein 4r1
101249671	No descriptions
101258116	No descriptions
101266793	No descriptions
101254502	No descriptions
101265846	No descriptions

SINLP6

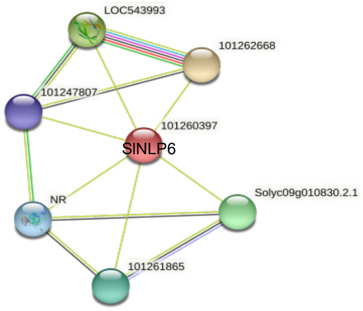

node	annotation
NR	Nitrate reductase
101262668	3-methylcrotonyl-CoA carboxylase
LOC543993	Biotin carboxyl carrier protein of acetyl-CoA carboxylase
Solyc09g010830.2.1	No descriptions
101261865	No descriptions
101247807	No descriptions
